# Impact of self-control and time perception on intertemporal choices in gain and loss situations

**DOI:** 10.3389/fpsyg.2023.1324146

**Published:** 2024-02-09

**Authors:** Weiguo Qu, Ying Yang, Mengjie Zhou, Wei Fan

**Affiliations:** ^1^Cognition and Human Behavior Key Laboratory of Hunan Province, Hunan Normal University, Changsha, China; ^2^Department of Psychology, School of Education Science, Hunan Normal University, Changsha, China; ^3^Institute of Interdisciplinary Studies, Hunan Normal University, Changsha, China

**Keywords:** gain-loss situation, self-control, time perception, intertemporal choice, moderating mediating effect

## Abstract

Individuals frequently encounter dilemmas in which they must choose between smaller, immediate gains and larger, delayed rewards; this phenomenon is known as intertemporal choice. The present study analyzed the interplay of trait and state self-control and time perception tendencies (time overestimation vs. time underestimation) and how it influences the rates of selecting immediate options in both gain and loss situations by conducting an intertemporal choice task. Experiment 1 was used to explore the impact of trait self-control and time perception on intertemporal choices within gain and loss situations. In Experiment 2, the e-crossing task was used to induce self-control resource depletion in participants and to investigate the impact of self-control resources and time perception on intertemporal choices in gain and loss situations. The results indicate that (1) compared with the high-self-control group, the low-self-control group exhibited a greater tendency to choose immediate options. Additionally, the high time estimation group was more likely to opt for immediate choices than the low time estimation group was. Furthermore, participants were more likely to select immediate options in the loss situation than in the gain situation. (2) In the gain situation, the high time estimation group was more likely to choose immediate options than was the low time estimation group. However, in the loss situation, the difference between the two groups was nonsignificant. (3) Time perception and gain–loss situations exerted a moderating mediating effect on the impact of self-control resources on intertemporal choices. These findings shed light on the influence of both self-control abilities and self-control resources on intertemporal choices. They provide valuable insights into intertemporal decision behaviors across diverse contexts and indicate the need for rational analysis based on one’s current state to mitigate cognitive biases to ensure individuals can maximize benefits in their daily lives.

## 1 Introduction

In their daily lives, individuals frequently encounter the dilemma of being required to choose between small rewards that they can gain immediately and larger rewards that they can obtain later. In such situations, individuals tend to prefer smaller, immediate gains over larger, delayed returns (Loewenstein and Prelec, 1992; Loewenstein, 1996; Kalenscher and Pennartz, 2008; Peters and Büchel, 2011). This phenomenon is referred to as intertemporal choice. When confronted with intertemporal choices, individuals must carefully consider the costs and benefits at different points in time, enabling them to form explicit judgments when making decisions (Frederick et al., 2002). Intertemporal choice has become a prominent area of research in the field of behavioral decision-making. It encompasses various aspects of life, including balancing immediate desires versus long-term health, making rational consumption choices, and planning financial investments (Reeck et al., 2017). Studies have indicated that intertemporal choice is affected by various factors, including individual factors, such as time perception, preferences, self-control, and temporary emotional states, and objective factors, such as choice attributes, delay durations, and gain–loss situations (Kim and Zauberman, 2009; Doidge et al., 2018; Malesza, 2019; Wang and He, 2020; Xu et al., 2020; Pei et al., 2022; Zhou et al., 2022). Psychological studies have revealed that individuals who often opt for delayed rewards in their daily lives tend to have better academic performance, greater career success, and a higher socioeconomic status (Moffitt et al., 2011; Gellert et al., 2012; Ishii, 2015). Moreover, the ability to delay rewards has been reported to be associated with enhanced adaptability, healthier habits, and a lower propensity to engage in addictive behaviors (Tangney et al., 2004; Reynolds, 2006; Daugherty and Brase, 2010). Self-control plays a pivotal role in intertemporal choice, profoundly affecting how individuals weigh current costs and benefits against future risks and rewards. Self-control involves restraining immediate gratification impulses and focusing on long-term objectives, which enables individuals to make more prudent choices. Self-control is one of the most critical and practical psychological attributes that an individual can develop, particularly when they face temptations (Turner et al., 2019).

Self-control abilities substantially affect individuals’ intertemporal choices. A study reported marked differences in intertemporal choices among individuals with varying levels of trait self-control (Liu et al., 2014). Compared with individuals with low levels of trait self-control, those with high levels of such self-control are more likely to adopt a long-term perspective, exhibit greater patience in their intertemporal choices, and select options that offer delayed but larger rewards. Hare et al. (2009) analyzed intertemporal choices by using a food choice task in which participants were required to choose between foods with varying levels of health benefits. The results revealed that individuals with high self-control were more likely to choose healthier foods, highlighting their tendency to prioritize long-term benefits when making intertemporal decisions. Most studies on intertemporal choices have focused on the effect of self-control abilities on intertemporal choices, overlooking variations in intertemporal choices among individuals with different self-control resources. Research on the impact of self-control resources on decision-making is primarily manifested in two aspects: decision styles and decision tendencies. Slovic et al. (2004) distinguished between two decision systems: the intuitive system and the deliberate system (Slovic et al., 2004). Previous study found that ego depletion can inhibit individuals’ deliberate decision system but does not affect the intuitive decision system (Pocheptsova et al., 2009). However, there is still controversy regarding the mechanisms through which ego depletion affects decision tendencies. Some researchers argued that ego depletion lead individuals to be more focused on the negative consequences of decisions, thereby triggering risk aversion (Unger and Stahlberg, 2011; Pohl et al., 2013). Another group of researchers suggested that when individuals were in a state of ego depletion, their self-control decreased sharply, making them more prone to favoring choices that offered immediate gratification (He and Yan, 2015; Guan and He, 2018; Malkoc and Zauberman, 2019). Suo et al. (2018) conducted a study in which they revealed that self-control abilities play a leading role in intertemporal decision-making and reported individual differences in the effect of self-control resources on decision-makers’ intertemporal choices. Therefore, expanding on previous research, the present study explored the effect of state self-control on intertemporal choices. Accordingly, we proposed Hypothesis 1: *Individuals with low levels of trait self-control tend to favor smaller immediate gains in intertemporal choices, whereas individuals with high levels of trait self-control are more likely to choose larger, delayed rewards.*

Studies have indicated that impulsivity may not be the only factor leading people to deviate from economic rationality. Some studies have reported that time perception may play a crucial role in decision-making and affect the formation of delay discounting behavior (Suo et al., 2014; Xu et al., 2020; Yang et al., 2021). Time perception involves subjective judgments regarding the duration or speed of time without reference to time-related cues or timing instruments (Huang et al., 2005). Han and Takahashi (2012) indicated that human intertemporal choices are considerably affected by the psychophysical aspects of time perception. Time perception follows Weber’s law, which states that subjective time is logarithmically related to objective time (Gibbon, 1977; Grondin, 2010). Furthermore, a study indicated that a close relationship exists between time discounting and time perception, with individuals exhibiting impulsivity in delay discounting often also experiencing disruptions in time estimation (Stam et al., 2022). In addition, intertemporal choices associated individuals’ time perception (Namboodiri et al., 2014; Xu et al., 2020). Reynolds and Schiffbauer (2004) revealed that compared with a control group with normal sleep, a sleep-deprived experimental group exhibited greater impulsivity, higher discounting of delayed rewards, and a substantial overestimation of time intervals. Studies on addiction have consistently indicated that individuals with several types of addiction (including drug, alcohol, and cigarette addiction) are more likely to impulsively choose immediate but smaller rewards when presented with intertemporal choices compared with healthy adults. Furthermore, their time perception abilities are often notably impaired (Bickel and Marsch, 2001; Reynolds and Schiffbauer, 2004; Ohmura et al., 2005; Sayette et al., 2005; Wittmann et al., 2007). In summary, individuals with heightened time perception abilities demonstrate a superior capacity for precise time estimation and engage in value assessments characterized by rationality and realism. Thus, they prioritize long-term objectives that promise enhanced value. By contrast, individuals with diminished time perception abilities struggle with accurate time estimation and thus prioritize short-term goals over long-term pursuits (Lukinova and Erlich, 2021; Zhou et al., 2022). Each individual’s subjective experience of time plays a pivotal role in their decision-making behavior. Subjective time perception exerts a more pronounced influence on decision-making than does objective time duration (Zauberman et al., 2009; Xu et al., 2020; Agostino et al., 2021). Suo et al. (2014) reported that time perception is crucial in intertemporal choices. In their study, regardless of the difficulty of a task, individuals who overestimated time were more likely to opt for smaller, immediate rewards, whereas those who underestimated time were more likely to opt for larger, delayed rewards. This finding can be attributed to overestimation of delay time causing decision-makers to overestimate the cost of waiting, resulting in more impulsive behavior in intertemporal choices, with a preference for immediate options (Wittmann and Paulus, 2008). Laube and van den Bos (2020) indicated that time estimation influences the impact of emotions on intertemporal choices. When individuals consider options associated with high positive outcomes, rather than those associated with low positive outcomes, they tend to estimate longer future durations. This finding indicates that individuals perceive delayed options to have reduced value, making them less enticing. Thus, people tend to choose more immediate but smaller rewards. On the basis of these findings, we proposed Hypothesis 2: *Individuals with heightened time perception tend to overestimate time and the time cost of delayed rewards, making them more likely to choose smaller, more immediate rewards. By contrast, individuals with diminished time perception tend to underestimate time and the time cost of delayed rewards, making them more likely to choose larger, delayed rewards.*

Studies have extensively investigated the association between self-control and time perception (Baird et al., 2017; Kim et al., 2017). According to self-control depletion theory, self-control is a finite resource that gradually becomes depleted after sustained engagement in self-control tasks. This depletion impairs subsequent self-control performance, rendering individuals more susceptible to self-control failure (Baumeister et al., 1998). Vohs and Schmeichel (2003) reported that depletion of self-control resources can alter an individual’s subjective perception of time. Individuals with strong self-control abilities often exhibit more accurate time perception. They can effectively estimate the time required to complete tasks, which aids them in time management and planning (Vohs and Schmeichel, 2003). Enhanced time perception can help individuals balance their current and future interests, facilitating long-term and rational decision-making (Diekhof et al., 2012; Suo et al., 2018). Droit-Volet and Dambrun (2019) determined that individuals with lower levels of state self-control tend to have impaired time duration estimation. This can lead to them becoming caught in a state of extending the present moment that eventually results in self-control failure and giving in to temptations. Voce and Moston (2016) experimentally explored the association between self-control and time perception. The differences in the intertemporal choices made by individuals with high and low levels of self-control are believed to be related to time perception. When presented with immediate, smaller gains and delayed, larger rewards, individuals with low self-control levels may overestimate time and consequently overestimate the costs of the delayed options and make impulsive choices (Suo et al., 2014; Takahashi, 2016; Kim et al., 2017). In summary, self-control levels can affect time perception. Individuals in a state of high self-control depletion expend more psychological resources, leading to low state self-control levels, inaccurate time perception, and a perception that time passes slowly (overestimating time). This in turn hampers their ability to effectively complete subsequent tasks, leading to failures. Accordingly, we proposed Hypothesis 3: *Self-control resources can indirectly predict intertemporal decisions through the mediating effect of time perception. Compared with the nonlossy group, the lossy group had lower self-control levels. Individuals with low levels of self-control overestimate time and the time cost of long-term returns and are more inclined to choose immediate options.*

In addition to the specific contributions of self-control and time perception to intertemporal choices, the influence of different decision contexts must be considered. In their daily lives, people are often faced with choices that can lead to them gaining rewards and choices that can lead to them avoiding losses. The framing of information can affect individuals’ choices within the same task, leading to reversals in decision preferences; this phenomenon is known as the framing effect (De Martino et al., 2006). Studies found that making decisions under gain and loss frames elicits distinct cognitive neural activities. Specifically, in the gain domain, the decision-making process involved central regions, including the medial prefrontal cortex and orbitofrontal cortex, while in the loss domain, it implicated the lateral dorsolateral prefrontal cortex (Gehring and Willoughby, 2002; Zhang et al., 2018). These findings suggest that intertemporal choices in gains and losses may engage different valuation systems, and more importantly, the neural interactions implementing these choices might be independent (Ohmura et al., 2005; Xu et al., 2009; Zhang et al., 2018). Individuals exhibit different time discount rates in different intertemporal decision contexts. Studies have reported that participants’ preference for immediate options is significantly lower in gain situations than in loss situations. According to prospect theory (Kahneman and Tversky, 1979), individuals exhibit loss aversion in gain situations and often demonstrate risk-seeking behavior in loss situations (Holt et al., 2008; Xu et al., 2009). This suggests that individuals are more inclined to be risk-averse when facing potential gains and tend to seek risks when confronted with potential losses. In gain contexts, when problems are presented in positive terms, decision-makers tend to choose immediate options. However, when problems are presented in negative terms, decision-makers tend to choose delayed options. In other words, individuals making decisions presented using gain framing tend to prefer immediate options. Regarding intertemporal choices with loss framing, individuals exhibit a tendency toward risk-seeking behavior, and individuals who overestimate time are more inclined to choose larger but delayed losses (Liu et al., 2012; Ma et al., 2012). Thus, we proposed Hypothesis 4: *Whether a situation is a gain or loss situation exerts a moderating effect on the influence of time perception on intertemporal decision-making.*

To test the aforementioned hypotheses, we designed two experiments. In Experiment 1, we investigated participants’ levels of trait self-control by using a self-control questionnaire. Subsequently, the present study analyzed the interplay of trait self-control and time perception tendencies and how it influences the rates of selecting immediate options in both gain and loss situations by conducting an intertemporal choice task. In Experiment 2, we induced depletion of participants’ self-control resources by using experimental methods and investigated the interaction between self-control resources and time perception in intertemporal choices within gain and loss contexts.

## 2 Experiment 1

### 2.1 Methods

#### 2.1.1 Participants

We estimated the minimum required sample size using G*Power (version 3.1.9.7) (Faul et al., 2009). With an *α* level of 0.05 and a power of 0.8, the simulation indicated that a sample size of *N* = 48 would be sufficient to detect a medium effect size *f* of 0.25. Based on the consideration of sample loss rate, we recruited 246 college students, who subsequently completed the Chinese version of Tangney’s Self-Control Scale (SCS; Tangney et al., 2004; Tan and Guo, 2008; Yin et al., 2022). Among the 246 participants, the 74 who scored among the highest 30% were included in the high self-control group, and the 74 who scored among the lowest 30% were included in the low self-control group (*M_high self-control_* = 69.49 ± 5.79, *M_low self-control_* = 43.66 ± 5.81; *t*(146) = −27.22, *p* < 0.001). Two participants were unable to participate in the experiment for personal reasons, with 146 remaining participating participants. Subsequently, we randomly assigned these participants (30 men and 116 women, aged between 18 and 26 years) to one of the following four groups: high self-control time overestimation group (*n* = 35; 11 men), high self-control time underestimation group (*n* = 38; 10 men), low self-control time overestimation group (*n* = 37; 5 men), and low self-control time underestimation group (*n* = 36, 4 men). All participants were right-handed and had normal or corrected-to-normal vision. None had psychological or neurological disorders or had previously participated in any similar study. After the experiment, participants received a basic reward of CNY 10.00, along with additional performance-related bonuses ranging from CNY 1 to CNY 10. This study was approved by the Local Ethics Committee of Hunan Normal University.

#### 2.1.2 Experimental design

In Experiment 1, we used a mixed experimental design involving a 2 (trait self-control: high vs. low) × 2 (time perception: time overestimation vs. time underestimation) × 2 (gain–loss situation: gain vs. loss) framework. Self-control ability and time perception were treated as between-subject variables, whereas the gain–loss situation was considered a within-subject variable. The dependent variable was the participants’ intertemporal decision outcomes, measured as the rate of choosing immediate options.

#### 2.1.3 Materials and experimental tasks

##### 2.1.3.1 Self-control scale

Tangney’s SCS is a self-report measure used to assess individual differences in trait self-control. A higher score indicates a greater capacity for self-control (Tangney et al., 2004). This scale comprises 19 items distributed across five dimensions: impulse control, healthy habits, resisting temptation, work focus, and entertainment moderation. A sample item is “I can resist temptation well” (resisting temptation). Items are rated on a 5-point Likert scale, with endpoints ranging from 1 (strongly disagree) to 5 (strongly agree). Items 1, 5, 11, and 14 are positively worded and scored in a straightforward manner, whereas the other items are negatively worded and reverse scored. A higher total score indicates a higher level of self-control. The Chinese version of the SCS was revised by Tan and Guo (2008), with this version having a Cronbach’s alpha of 0.862 and a test–retest reliability of 0.850 (measured over a 3-week interval). The Cronbach’s alpha for the SCS among our participants was 0.884.

##### 2.1.3.2 Time reproduction task

**Figure 1:**
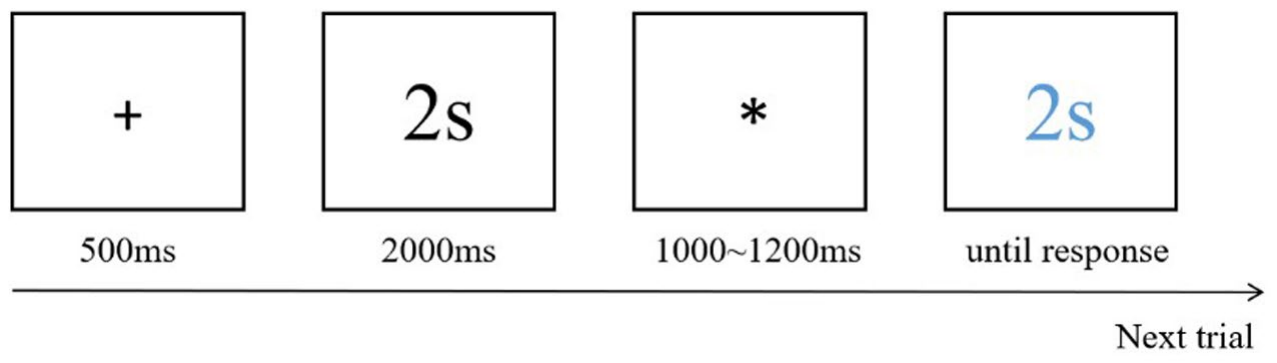
One trial of the time reproduction task.

This task was used to investigate the participants’ perception and estimation abilities regarding time (Mioni et al., 2014, 2016; Suo et al., 2014; Yin et al., 2023). In this task, participants are required to estimate the duration of a specific time interval, typically by observing a stimulus (such as a displayed number) that is presented for a certain period, and then asked to accurately reproduce that duration. In the present study, this task was used to assess the participants’ subjective perception of the passage of time and their ability to accurately estimate the length of time. The task commenced with the presentation of a fixation point for 500 ms, which signaled the start of the experiment. Subsequently, a black number (e.g., 2, 4, 8, and 16) appeared on the screen, with the black number being equal to the number of seconds it remained visible for. An asterisk “*” was randomly presented in the center of the screen, with the duration of its visibility varying between 1,000 and 1,200 ms. After, a blue number identical to the previous black number appeared in the center of the screen. The participants were asked to press the “enter” key when they believed that the blue number had been visible for the number of seconds it represented. They then proceeded to the next trial (Figure 1). Three practice trials were conducted before the formal experiment commenced.

##### 2.1.3.3 The intertemporal choice paradigm

In this experiment, we employed the intertemporal choice paradigm introduced by Chen and He (2011). Prior to the experiment, the participants were explicitly informed that they would be engaging in a real decision-making task with the opportunity to receive monetary rewards. In addition, the participants were told that probe options would intermittently appear throughout the experiment to verify their engagement in each decision round. They were informed that only those who completed the entire experiment and remained engaged throughout would be eligible to receive the rewards. We used a questionnaire in which the participants were asked to indicate their preferences regarding 2 scenarios by responding to 19 items for each scenario; each item presented two options: Option A and Option B. For each item, the time and amount associated with Option B remained fixed. Option A had a fixed time duration that remained constant across items, but the monetary amount increased incrementally with each item, with the amount starting at CNY 50 for the first item and increasing by CNY 50 for each subsequent item, with the amount reaching CNY 950 by the 19th item. The participants were asked to make choices for each item on the basis of their genuine preferences. Participants received two types of compensation: a basic fee for participating in the experiment and an additional fee determined by their performance in intertemporal decision-making. After the experiment, participants randomly drew one trial each in the gain and loss scenarios (calculated at a 1% rate) as the final additional participant fee. In the gain scenario, if the chosen option was immediate, participants received the corresponding amount immediately. If the chosen option was delayed (after 6 months), considering practical considerations, participants received the amount one week later. In the loss scenario, if the chosen option was immediate, participants immediately incurred the corresponding loss. If the chosen option was delayed, participants incurred the loss (paid to the researcher) one week later. This process was conducted using a pen-and-paper measurement method. The following presents the created profit and loss scenarios.

Please read the following carefully and select your preference:

Imagine that you have a part-time job, and given your outstanding performance, your boss decides to reward you with an additional bonus. There are two options for how this bonus can be paid out. One option is to receive it immediately, but the amount at this time is somewhat small. The other option is to receive it after a delay, but the bonus amount will be larger. Please choose your preference for each of the following options:

A. Receive CNY 50 immediately, B. Receive CNY 1000 after 6 months.

A. Receive CNY 100 immediately, B. Receive CNY 1000 after 6 months.

...

A. Receive CNY 950 immediately, B. Receive CNY 1000 after 6 months.

Imagine the following scenario: You are working part-time, and due to a mistake you made, your boss incurred a loss. You are required to take partial responsibility for the loss, and there are two options for how you can fulfill this responsibility. One option is to make an immediate payment, paying a smaller amount of money. The other option is to make a payment 6 month later, but it will require you to pay a larger compensation amount. Please make your choice from the following options:

A. Pay CNY 950 immediately, B. Pay CNY 1000 after 6 months.

A. Pay CNY 900 immediately, B. Pay CNY 1000 after 6 months.

...

A. Pay CNY 50 immediately, B. Pay CNY 1000 after 6 months.

On the basis of the description provided for each item, carefully consider and choose the option that best aligns with your personal preferences.

#### 2.1.4 Procedure

All participants completed the experiment in separate small rooms, and the experiment comprised three main parts. Firstly, the participants responded to the self-control ability scale and on the basis of their scores were divided into high and low trait self-control groups. Secondly, the participants completed the time reproduction task. Stimuli were presented, and behavioral data were collected using E-prime 2.0 software (Psychology Software Tools, Pittsburgh, PA, United States). According to the time reproduction task, the high trait self-control group was further divided into time overestimation and time underestimation groups, while the low trait self-control group was divided into time overestimation and time underestimation groups. To ensure that all participants understood the experimental procedure, practice trials were conducted before the formal experiment commenced. Finally, all participants completed the intertemporal choice task. They were informed that the experiment involved two real-life scenarios, each with 19 sets of options. We instructed them to carefully note the differences between the options and make their choices after weighing the options thoroughly.

#### 2.1.5 Data recording and analysis

Behavioral data were analyzed using SPSS 23.0 (IBM, Armonk, NY, USA). The independent-samples *t* test [2 (trait self-control: high vs. low)] was performed to compare the scores of the SCS under different conditions. The independent-samples *t* test [2 (time perception: time overestimation vs. time underestimation)] was also performed to compare the reaction times for time perception under different conditions. Analysis of variance (ANOVA) [2 (trait self-control: high vs. low) × 2 (time perception: time overestimation vs. time underestimation) × 2 (gain–loss situation: gain vs. loss)] was performed to compare the rate of choosing immediate options between different conditions.

### 2.2 Results

#### 2.2.1 Common method variance test

To address potential common method variance resulting from our use of self-report scales for data collection (Zhou and Long, 2004), we conducted Harman’s single-factor test for an assessment of the validity of the measurement results. Exploratory factor analysis was performed for all measured data. The results revealed that the initial eigenvalues of the three factors were all greater than 1. Furthermore, the proportion of variance explained by the first factor was 32.83%, which is less than the critical threshold of 40%. These findings indicate no substantial common method bias was present in this study.

#### 2.2.2 Manipulation check

##### 2.2.2.1 Manipulation check for trait self-control

The results of the independent-samples *t* test revealed that the scores on the SCS were higher in the high self-control group (M ± SD = 69.49 ± 5.79) than those in the low self-control group [M ± SD = 43.66 ± 5.81, *t*(146) = −27.22, *p* < 0.001, Cohen’s *d* = 4.45, 95% CI = (−27.73, −23.94)].

##### 2.2.2.2 Manipulation check for time perception

We grouped the participants on the basis of their performance in the time reproduction task. We sorted all participants in ascending order according to their total scores for time perception, with those scoring in the top 50% included in the time underestimation group and those scoring in the bottom 50% included in the time overestimation group. The results of the independent-samples *t* test revealed that the scores for time perception were higher in the time overestimation group (*M* ± *SD* = 31646.46 ± 3395.20) than in the time underestimation group [*M* ± *SD* = 26637.96 ± 3583.01, *t*(144) = −8.67, *p* < 0.001, Cohen’s *d* = 1.43, 95% CI = (−6150.96, −3866.04); Table 1].

**Table 1:** Differences in time perception scores among different groups in Experiment 1 (*M* ± *SD*).

	time underestimation group	time overestimation group	*t*	*p*	95% CI
	*M ± SD* (*n* = 74)	*M ± SD* (*n* = 72)			
2,000 ms	1822.11 ± 519.86	2186.40 ± 365.58	−4.89	< 0.001	[−511.67, −216.91]
4,000 ms	3502.23 ± 499.56	4166.23 ± 599.82	−7.28	< 0.001	[−844.37, −483.62]
8,000 ms	7006.37 ± 975.65	8280.07 ± 1086.77	−7.46	< 0.001	[−1611.35, −936.06]
16,000 ms	14307.35 ± 2405.18	17013.93 ± 1998.88	−7.38	< 0.001	[−3431.05, −1982.10]
Total	26637.96 ± 3583.01	31646.46 ± 3395.20	−8.67	< 0.001	[−6150.96, −3866.04]

Mean ± standard deviation, (M ± SD).

#### 2.2.3 Rate of choosing immediate options

**Figure 2:**
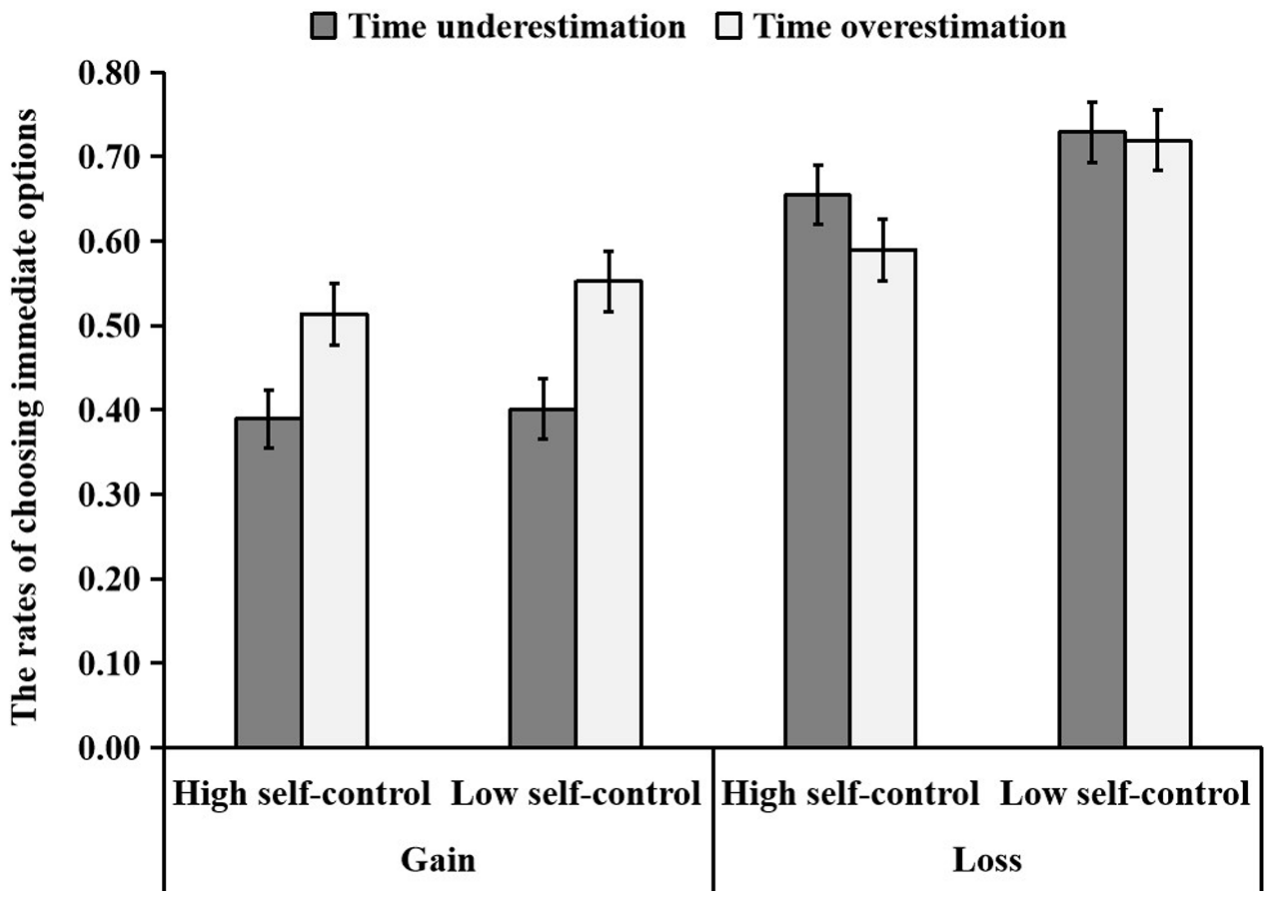
The difference in the ratio of self-control ability and time perception to choose the immediate option in the gain/loss situation. Error bars indicate standard errors of the mean.

The 2 (trait self-control: high vs. low) × 2 (time perception: time overestimation vs. time underestimation) × 2 (gain–loss situation: gain vs. loss) three-way ANOVA revealed trait self-control to have a significant main effect [*F* (1, 142) = 7.05, *p* = 0.009 $,\eta_{p}^{2}=$ 0.047]. The rate of choosing immediate options was higher in the low self-control group (*M* ± *SD* = 0.60 ± 0.23) than in the high self-control group [*M* ± *SD* = 0.54 ± 0.26, *p* = 0.009, 95% CI = (−0.11, −0.02)]. Time perception had a significant effect [*F* (1, 142) = 4.27, *p* = 0.041 $,\eta_{p}^{2}=$ 0.029]. The rate of choosing immediate options was higher in the time overestimation group (*M* ± *SD* = 0.59 ± 0.25) than in the time underestimation group [*M* ± *SD* = 0.54 ± 0.25, *p* = 0.041, 95% CI = (−0.10, −0.01)]. The gain–loss situation had a significant effect [*F* (1, 142) = 60.60, *p* < 0.001 $,\eta_{p}^{2}=$ 0.299]. The rate of choosing immediate options was higher in the loss situation (*M* ± *SD* = 0.67 ± 0.21) than in the gain situation [*M* ± *SD* = 0.46 ± 0.24, *p* < 0.001, 95% CI = (−0.26, −0.16); Figure 2].

The interaction between time perception and gain–loss situation was significant [*F*(1,142) = 10.72, *p* < 0.001 $,\eta_{p}^{2}$ = 0.070]. Furthermore, a simple effect analysis revealed a significant difference in the rate of choosing immediate options among the different time perception groups in the gain situation [*F*(1,142) = 13.60, *p* < 0.001 $,\eta_{p}^{2}$ = 0.087], with the results indicating that the rate of choosing immediate options was higher in the time overestimation group (*M* ± *SD* = 0.53 ± 0.26) than in the time underestimation group [*M* ± *SD* = 0.39 ± 0.18, *p* < 0.001, 95% CI = (−0.21, −0.06)] in the gain situation. However, the rate of choosing immediate options did not differ between the time overestimation group and the time underestimation group in the loss situation [*F*(1,142) = 1.22, *p* = 0.271 $,\eta_{p}^{2}$ = 0.009]. The interaction between trait self-control and time perception was nonsignificant [*F*(1,142) = 0.74, *p* = 0.390 $,\eta_{p}^{2}$ = 0.005]. In addition, the interaction between trait self-control and gain–loss situation was nonsignificant [*F*(1,142) = 2.09, *p* = 0.151 $,\eta_{p}^{2}$ = 0.014], and the three-factor interaction term of trait self-control × time perception × gain–loss situation was nonsignificant [*F*(1,142) = 0.07, *p* = 0.799 $,\eta_{p}^{2}$ = 0.001; Figure 2].

### 2.3 Discussion of experiment 1

The results of Experiment 1 indicate that the participants with high levels of trait self-control exhibited a lower rate of choosing immediate options than did those with low levels of trait self-control. Hypothesis 1 was confirmed. This finding is consistent with those of previous studies (Hare et al., 2009; Malkoc and Zauberman, 2019; He et al., 2020). Trait self-control is a stable personality trait that affects various aspects of an individual’s behavior (Turner et al., 2019). Individuals with high levels of trait self-control are adept at controlling their impulses and resisting temptations when pursuing goals and making decisions, enabling them to exercise self-restraint and commitment (Tangney et al., 2004). Individuals with high trait self-control tend to opt for larger but delayed rewards to secure greater benefits (Seaman et al., 2018). Moreover, the present study’s results further revealed that the participants who overestimated time were more inclined to choose immediate options in intertemporal choices. This finding aligns with that of a previous study (Suo et al., 2014). Compared with the participants who underestimated time, those who overestimated time perceived a longer waiting time for delayed rewards. Prolonged waiting periods are often associated with increased risks and greater uncertainty, and therefore, individuals who overestimate time perceive delayed rewards to have lower subjective value. The aformentioned finding confirms Hypothesis 2. In gain situations, the participants of this study tended to favor larger, delayed rewards over smaller, immediate gains. By contrast, in loss situations, a preference reversal was observed, with the participants favoring smaller, immediate losses over larger, delayed ones. These findings are in line with those of previous studies demonstrating similar preference shifts over time (Ma et al., 2012; Zhang et al., 2018). The participants of this study exhibited different preferences in their intertemporal choices in gain and loss situations (Xu et al., 2022); this finding supports Hypothesis 3.

The results of this study indicated that in gain situations, the participants who overestimated time were more likely to choose immediate options than were those who underestimated time. However, in loss situations, no significant difference was noted between these groups. Previous studies have indicated that the effect of gain–loss situations on intertemporal choices varies with an individual’s time perception (Zhang et al., 2018). This variability can be attributed to the stronger emotional experience and greater impact of negative information in loss situations rendering individuals more sensitive and alert to such contexts (Zhang et al., 2018). Furthermore, the brain is more sensitive to losses than to gains. Studies in the field of neuroscience have reported that activation of the brain regions associated with negative emotions, including the insula, thalamus, and posterior striatum, is stronger in loss situations than in gain situations (Zhang et al., 2018). This indicates an asymmetry is present in the neural mechanisms underlying gain and loss in intertemporal choices, with this asymmetry potentially driven by negative emotions, such as aversion.

## 3 Experiment 2

### 3.1 Method

#### 3.1.1 Participants

We estimated the minimum required sample size using G*Power (version 3.1.9.7) (Faul et al., 2009). With an *α* level of 0.05 and a power of 0.8, the simulation indicated that a sample size of *N* = 48 would be sufficient to detect a medium effect size *f* of 0.25. The selection of effect sizes was based on previous studies (Guan and He, 2018; Pei et al., 2022). Thus, we recruited 61 participants (27 men and 34 women, aged between 18 and 26 years). All participants were divided into four groups: Non-depletion time underestimation group (*n* = 15, 7 men), Non-depletion time overestimation group (*n* = 15, 10 men), Depletion time underestimation group (*n* = 15, 5 men), and Depletion time overestimation group (*n* = 16, 5 men). All participants were right-handed and had either normal or corrected-to-normal vision. None had psychological or neurological disorders or had previously participated in a similar study. After the experiment, participants received a basic reward of CNY 10.00, along with additional performance-related bonuses ranging from CNY 1 to CNY 10. This study was approved by the Local Ethics Committee of Hunan Normal University.

#### 3.1.2 Experimental design

In Experiment 2, we employed a 2 (self-control resource: depletion vs. nondepletion) × 2 (time perception: time overestimation vs. time underestimation) × 2 (gain–loss situation: gain vs. loss) mixed experimental design. Self-control resources and time perception were considered between-subject variables, whereas the gain–loss situation was treated as a within-subject variable. The dependent variable was the participants’ intertemporal decision outcomes, measured as the rate of choosing immediate options.

#### 3.1.3 Materials and experimental task

##### 3.1.3.1 The e-crossing task

This task was used to manipulate the participant’s self-control resources (Wheeler et al., 2007; Clarkson et al., 2010). The participants were provided with two English articles. Both the depletion and the nondepletion group received articles with the same content but different instructions. The e-crossing task consisted of two phases. In the first phase, participants from both the depletion and nondepletion groups were presented with the same instructions, being instructed to cross out all the letters “e” in the English article. In the second phase, participants in the two groups were presented with different instructions. The depletion group’s instruction was to “cross out the letter “e” but if the letter “e” was preceded or followed by two vowels in a word, then not to cross it out. For example, in words like “heat” or “make”, the letter “e” should not be crossed out. The time given was 5 min.” The nondepletion group’s instruction was to “crossed out all the letter “e” in this English article. The time given was 5 min.” Therefore, participants in the depletion group were required to cross out the letter “e” in the English articles based on specific rules (different instructions for the two phases). Participants in the nondepletion group were instructed to cross out the letter “e” based on the same rules (consistent instructions for the two phases). After the completion of the letter-crossing task, the participants were asked to complete a brief questionnaire, where they rated the task’s difficulty, their fatigue level, the effort expended, and the degree of energy depletion caused by the task. Participants responded to the following questions: How did they perceive the difficulty of the task? (1 = extremely easy, 7 = extremely difficult), how fatigued they felt (1 = not tired at all, 7 = very tired), the effort they put into the test (1 = not at all, 7 = very much), and the degree of energy depletion experienced (1 = not at all, 7 = very much). Ratings were provided on a 7-point scale, with higher scores indicating a stronger experience in each dimension. These scores were used as a quantitative measure to assess whether ego depletion was successfully induced in the participants across these four dimensions.

##### 3.1.3.2 Time reproduction task

The procedure for this task was the same as that in Experiment 1.

##### 3.1.3.3 The intertemporal choice paradigm

**Figure 3:**
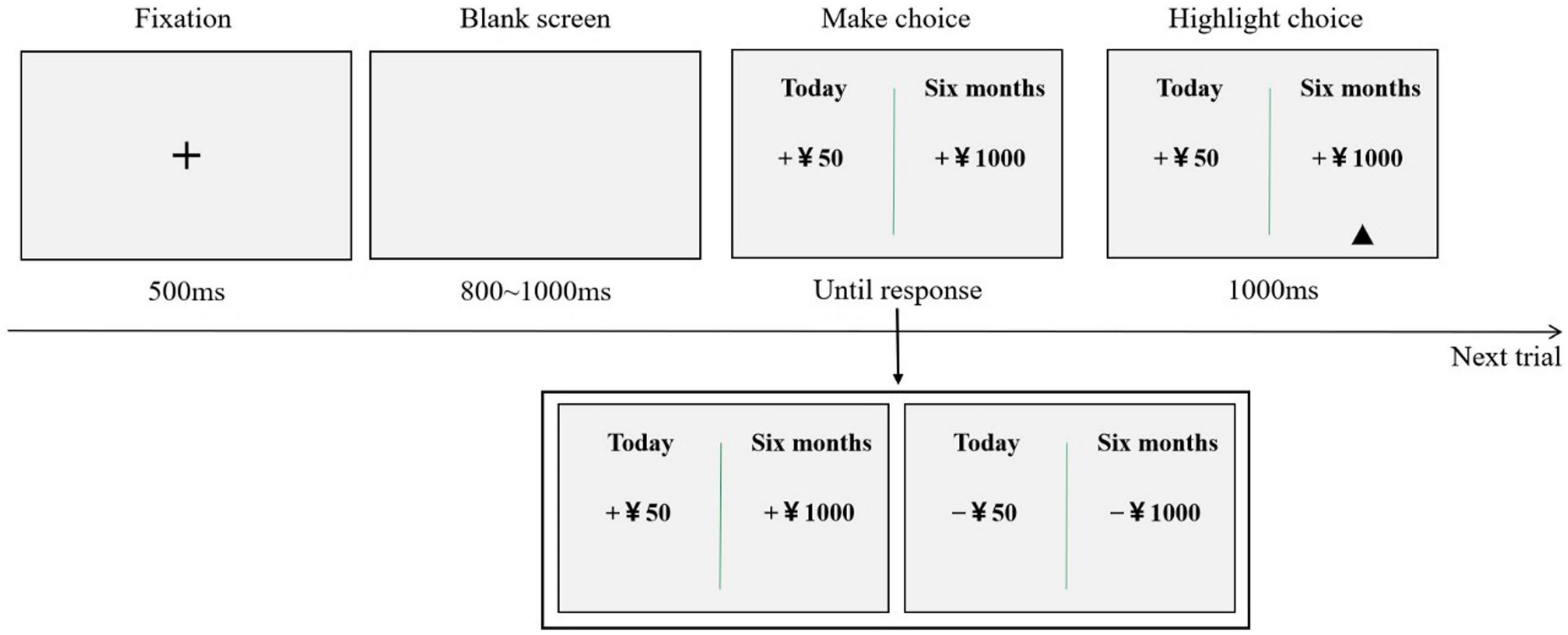
One trial of the intertemporal choice paradigm.

The procedure for this task was the same as that for Experiment 1. However, in Experiment 2, we employed a computer-based interface (Figure 3). Firstly, an initial fixation point (+) appeared at the center of the computer screen for 500 ms, serving as a cue for the participants to prepare for the experiment. Subsequently, two options were presented on the screen: the left option denoted an immediate choice and the right option represented a delayed choice. The participants were instructed to make selections on the basis of their genuine preferences. The participants were required to press the “F” key to choose the left option and the “J” key to choose the right option. After the participants pressed the key, a small triangle appeared below the chosen option for 1,000 ms to confirm the selection before the next trial commenced. The entire task was divided into 2 blocks, with each block consisting of 19 trials.

#### 3.1.4 Procedure

All participants completed the experiment in separate small rooms, and the experiment comprised three main segments. Firstly, the participants completed the e-crossing task and provided ratings for their perceived task difficulty, fatigue level, effort expended, and degree of energy depletion across four dimensions. We initially categorized participants into depletion group and nondepletion group. Secondly, according to the time reproduction task, the depletion group was further divided into time overestimation and time underestimation groups, while the nondepletion group was divided into time overestimation and time underestimation groups. Finally, all participants completed the intertemporal choice task. For both the time reproduction task and the intertemporal choice task, behavioral data were collected using E-prime 2.0 software (Psychology Software Tools, Pittsburgh, PA, United States). To ensure that all participants understood the experimental procedure, practice trials were conducted before the formal experiment commenced. The participants were informed that the experiment involved two real-life scenarios, each featuring 19 sets of options. They were instructed to carefully note the differences between the options and make their choices after weighing the options thoroughly.

#### 3.1.5 Data recording and analysis

Behavioral data were analyzed using SPSS 23.0 (IBM, Armonk, NY, United States). The independent-samples *t* test [2 (self-control resource: depletion vs. nondepletion)] was performed to compare the ego-depletion scores in different conditions. The independent-samples *t* test [2 (time perception: time overestimation vs. time underestimation)] was also performed to compare reaction times for time perception in different conditions. ANOVA [2 (self-control resource: depletion vs. nondepletion) × 2 (time perception: time overestimation vs. time underestimation) × 2 (gain–loss situation: gain vs. loss)] was performed to compare the rate of choosing immediate options between different conditions.

### 3.2 Results

#### 3.2.1 Manipulation check

##### 3.2.1.1 Manipulation check for self-control resources

The results of the independent-samples *t* test revealed that the score for the degree of ego depletion was higher in the depletion group (*M* ± *SD* = 15.74 ± 5.07) than that in the nondepletion group [*M* ± *SD* = 12.17 ± 4.76, *t*(59) = −2.84, *p* = 0.006, Cohen’s *d* = 0.73, 95% CI = (−6.10, −1.06); Table 2].

**Table 2:** Differences in time perception scores among different groups in Experiment 1 (*M* ± *SD*).

	The depletion group	The nondepletion group	*t*	*p*	Cohen’s *d*
	*M* ± *SD* (*n* = 31)	*M* ± *SD* (*n* = 30)			
Task difficulty	3.48 ± 1.36	2.77 ± 1.41	−2.02	0.048	0.51
Fatigue level	4.13 ± 1.34	3.07 ± 1.51	−2.92	0.005	0.74
Effort expended	4.16 ± 1.61	3.20 ± 1.32	−2.54	0.014	0.65
Energy depletion	3.97 ± 1.40	3.13 ± 1.31	−2.40	0.019	0.62
Total	15.74 ± 5.07	12.17 ± 4.76	−2.84	0.006	0.73

Mean ± standard deviation, (M ± SD).

##### 3.2.1.2 Manipulation check for time perception

We grouped the participants on the basis of their performance in the time reproduction task. We sorted the participants in ascending order according to their total time perception scores, with those scoring in the top 50% included in the time underestimation group and those in the bottom 50% included in the time overestimation group. The findings of the independent-samples *t* test revealed that the time perception score was higher in the time overestimation group (*M* ± *SD* = 32224.62 ± 3028.28) than that in the time underestimation group [*M* ± *SD* = 27432.71 ± 2338.10, *t*(59) = −6.90, *p* < 0.001, Cohen’s *d* = 1.77, 95% CI = (−6181.27, −3402.56); Table 3].

**Table 3:** Differences in time perception scores between different groups in Experiment 2 (*M* ± *SD*).

	time underestimation group	time overestimation group	*t*	*p*	95% CI
	*M ± SD* (*n* = 74)	*M ± SD* (*n* = 72)			
2000 ms	1765.93 ± 332.16	2180.67 ± 426.75	−4.23	< 0.001	[−611.12, −218.37]
4,000 ms	3500.42 ± 384.05	4346.84 ± 754.27	−5.50	< 0.001	[−1154.66, −538.18]
8,000 ms	7330.91 ± 681.62	8489.47 ± 1311.10	−4.31	< 0.001	[−1696.64, −620.48]
16,000 ms	14835.46 ± 1583.53	17207.65 ± 1238.12	−6.53	< 0.001	[−3099.10, −1645.27]
Total	27432.71 ± 2338.10	32224.62 ± 3028.28	−6.90	< 0.001	[−6181.27, −3402.56]

Mean ± standard deviation, (M ± SD).

#### 3.2.2 Rate of choosing immediate options

**Figure 4:**
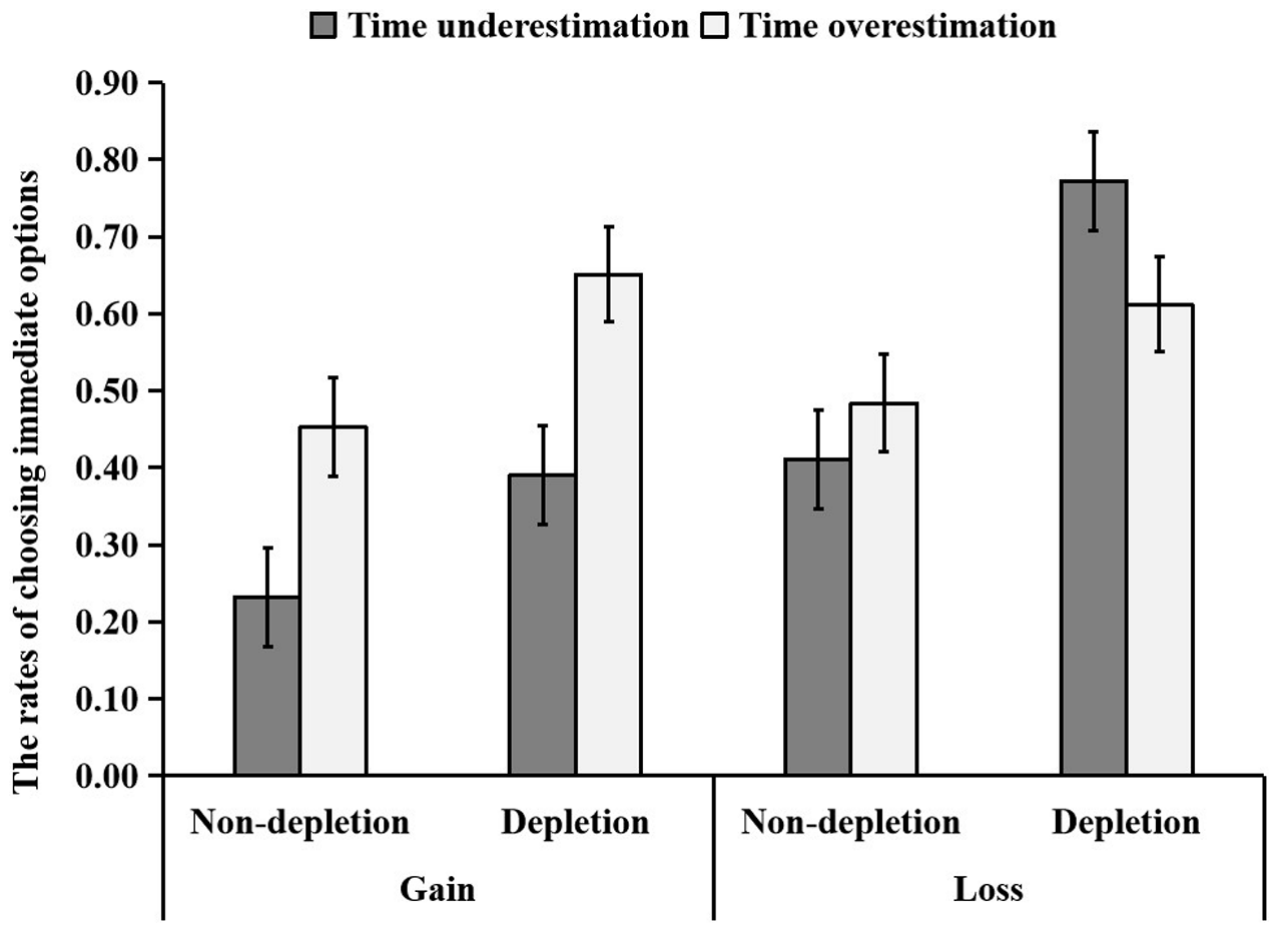
The difference in the ratio of self-control resource and time perception to choose the immediate option in the gain/loss situation. Error bars indicate standard errors of the mean.

The 2 (self-control resource: depletion vs. nondepletion) × 2 (time perception: time overestimation vs. time underestimation) × 2 (gain–loss situation: gain vs. loss) three-way ANOVA revealed self-control resources to have a significant main effect of [*F*(1, 57) = 23.36, *p* < 0.001 $,\eta_{p}^{2}$ = 0.291]. The rate of choosing immediate options was higher in the depletion group (*M* ± *SD* = 0.61 ± 0.26) than that in the nondepletion group [*M* ± *SD* = 0.40 ± 0.28, *p* < 0.001, 95% CI = (−0.30, −0.12)]. Time perception had a significant effect [*F* (1, 57) = 5.14, *p* = 0.027 $,\eta_{p}^{2}=$ 0.083]. The rate of choosing immediate options was higher in the overestimating time group (*M* ± *SD* = 0.55 ± 0.27) than that in the underestimating time group [*M* ± *SD* = 0.45 ± 0.30, *p* = 0.027, 95% CI = (−0.19, −0.01)]. The gain–loss situation had a significant effect [*F* (1, 57) = 8.98, *p* = 0.004 $,\eta_{p}^{2}=$ 0.136]. The rate of choosing immediate options was higher in the loss situation (*M* ± *SD* = 0.57 ± 0.27) than that in the gain situation [*M* ± *SD* = 0.43 ± 0.29, *p* = 0.004, 95% CI = (−0.23, −0.05); Figure 4].

The interaction between time perception and gain–loss situation was significant [*F*(1, 57) = 9.50, *p* = 0.003, $\eta_{p}^{2}$ = 0.143]. Furthermore, the results of simple effect analysis revealed a significant difference in the rate of choosing immediate options between the different time perception groups in the gain situation [*F*(1, 57) = 13.56, *p* = 0.001, $\eta_{p}^{2}$ = 0.192], with the results indicating that the rate of choosing immediate options was higher in the time overestimation group (*M* ± *SD* = 0.55 ± 0.29) than that in the time underestimation group [*M* ± *SD* = 0.31 ± 0.24, *p* < 0.001, 95% CI = (−0.21, −0.06)] in the gain situation. However, the rate of choosing immediate options did not differ between the time overestimation group and the time underestimation group in the loss situation [*F*(1, 57) = 0.49, *p* = 0.486 $,\eta_{p}^{2}$ = 0.009]. The interaction between self-control resources and time perception was nonsignificant [*F*(1, 57) = 1.22, *p* = 0.275 $,\eta_{p}^{2}$ = 0.021]. The interaction between self-control resources and gain–loss situation was nonsignificant [*F*(1, 57) = 0.51, *p* = 0.476, $\eta_{p}^{2}$ = 0.009]. The three-factor interaction term of trait self-control × time perception × gain–loss situation was nonsignificant [*F*(1, 57) = 2.21, *p* = 0.143 $,\eta_{p}^{2}$ = 0.037; Figure 4).

#### 3.2.3 Testing moderated mediation model

The ANOVA results revealed that self-control resources and time perception significantly affected the rate of selecting immediate options in intertemporal choices. This finding provided the foundation for our mediation analysis. We standardized the variables related to self-control resources, time perception, gain–loss situation, and intertemporal choices. The self-control resource score was the total score of the four dimensions: task difficulty, fatigue level, effort expended, and energy depletion. Time perception was the total score of perceived durations for four time intervals: 2,000 ms, 4,000 ms, 8,000 ms, and 16,000 ms. Subsequently, following the approach proposed by Wen and Ye (2014), we conducted a three-step investigation of the moderated mediation model.

**Figure 5:**
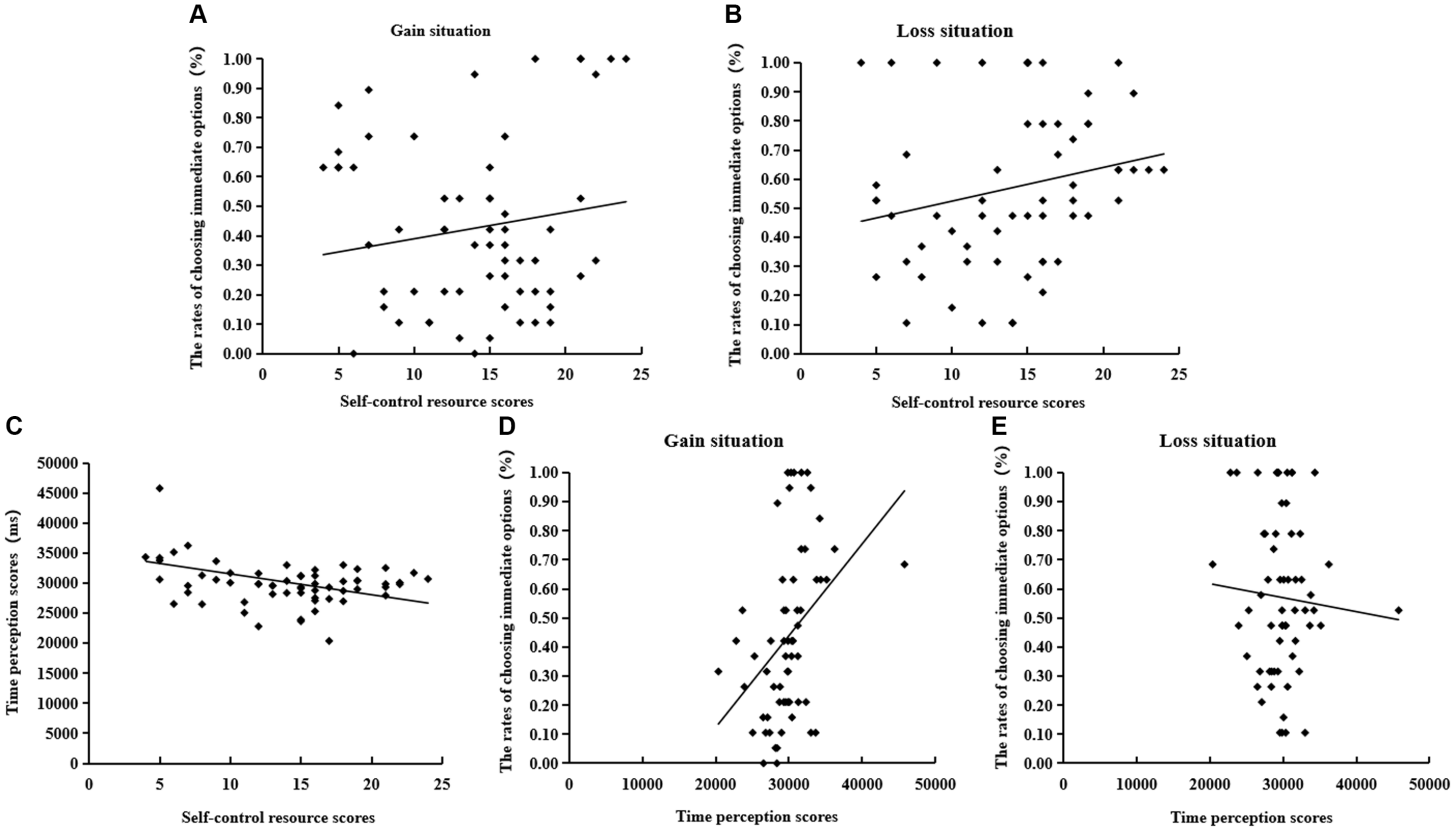
Panel **(A)**: the scatter plot depicts self-control resource scores and the rates of immediate options in the gain situation; Panel **(B)**: the scatter plot depicts self-control resource scores and the rates of immediate options in the loss situation; Panel **(C)**: the scatter plot depicts self-control resource scores and time perception scores; Panel **(D)**: the scatter plot depicts time perception scores and the rates of immediate options in the gain situation; Panel **(E)** the scatter plot depicts time perception scores and the rates of immediate options in the loss situation.

In the first step, we investigated the predictive effect of self-control resources on time perception. Using SPSS, we performed a regression analysis by using self-control resources as the independent variable and time perception as the dependent variable. The results revealed a significant predictive effect of self-control resources on time perception (*β =* −0.29, *SE* = 0.09, *p* = 0.001) (see Figure 5C). The adjusted model yielded the following results [*R*^2^ = 0.09, △*R*^2^ = 0.08, *F*(1, 120) = 10.85, *p* = 0.001].

In the second step, we analyzed the mediating effect of time perception on the association between self-control resources and intertemporal choices by using Model 4 from the PROCESS plugin. We employed self-control resources as the independent variable, time perception as the mediating variable, and intertemporal choice as the dependent variable. The results revealed that self-control resources exerted a significant predictive effect on intertemporal choice (*β =* 0.20, *SE* = 0.09, *p* = 0.029) (see Figures 5A,B). Furthermore, time perception had a significant predictive effect on intertemporal choice (*β =* 0.23, *SE* = 0.09, *p* = 0.016; as illustrated Model 4 in Table 4) (see Figures 5D,E).

**Table 4:** Results of moderated mediation model test (*N* = 122).

Process	Variables	Model 4				Model 14			
		*β*	*SE*	*t*	95% CI	*β*	*SE*	*t*	95% CI
1. Me	X	−0.29	0.09	−3.29^**^	[−0.46, −0.11]	−0.29	0.09	−3.29^**^	[−0.46, −0.11]
2. Y	X	0.20	0.09	2.21^*^	[0.02, 0.39]	0.20	0.09	2.33^*^	[0.03, 0.38]
	Me	0.23	0.09	2.44^*^	[0.04, 0.41]	0.90	0.27	3.39^***^	[0.38, 1.43]
	Mo					0.47	0.17	2.8^**^	[0.14, 0.80]
	Me*Mo					−0.45	0.17	−2.69^**^	[−0.79, −0.12]
*R*^2^ = 0.07, *F*(df) = 4.22 (119)						*R^2^ =* 0.17, *F*(df) = 6.11 (117)			

*X*, self-control resource; Me, time perception; Mo, gain–loss. gain–loss situation, Y, Rate of choosing immediate options; 95% confidence intervals for predictor variables were obtained using the bootstrap method; *p < 0.05, **p < 0.01, ***p < 0.001.

In the third step, we investigated the moderating effect of the gain–loss situation on the mediating model. Using SPSS 23.0 with the PROCESS plugin and following the bootstrap method proposed by Hayes (2013), we selected Model 14. With a sample size of 5,000 and a 95% confidence interval, we used self-control resources as the independent variable (X), the rate of choosing the immediate option as the dependent variable (Y), time perception as the mediating variable (M), and the gain–loss situation as the moderating variable (V). The results indicated that self-control resources had a significant predictive effect on intertemporal choices (*β =* 0.20, *SE* = 0.09, *p* = 0.021). Time perception significantly predicted intertemporal choices (*β =* 0.90, *SE* = 0.27, *p* = 0.001). The gain–loss situation had a significant predictive effect on intertemporal choices (*β =* 0.47, *SE* = 0.17, *p* = 0.006). The interaction between time perception and the gain–loss situation had a significant predictive effect on intertemporal choices (*β =* −0.45, *SE* = 0.17, *p* = 0.008). These findings indicate that the gain–loss situation plays a moderating role in the effect of self-control resources on intertemporal choices through time perception, and it moderates the latter part of the model’s pathway (as illustrated in Model 14 of Table 4).

situation, Y = Rate of choosing immediate options; 95% confidence intervals for predictor variables were obtained using the bootstrap method; ^*^p < 0.05, ^**^p < 0.01, ^***^p < 0.001.

**Figure 6:**
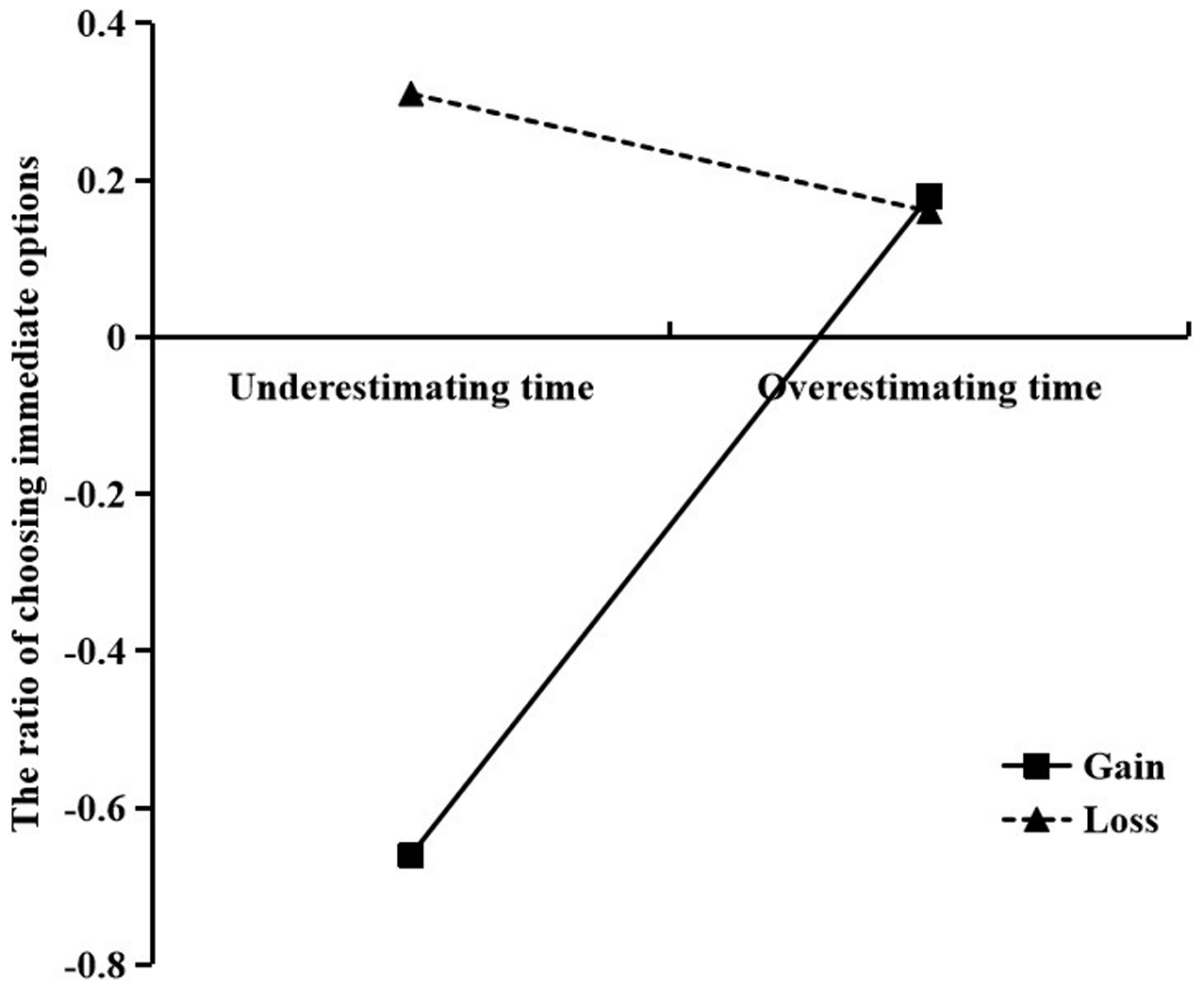
The moderating effect of gain-loss situation on the influence of time perception on intertemporal choices.

To investigate the moderating role of the gain–loss situation, following the suggestion of Cohen et al. (2003), we plotted simple slopes to illustrate the association between time perception and the rate of choosing immediate options in the gain–loss situation (Figure 6).

**Figure 7:**
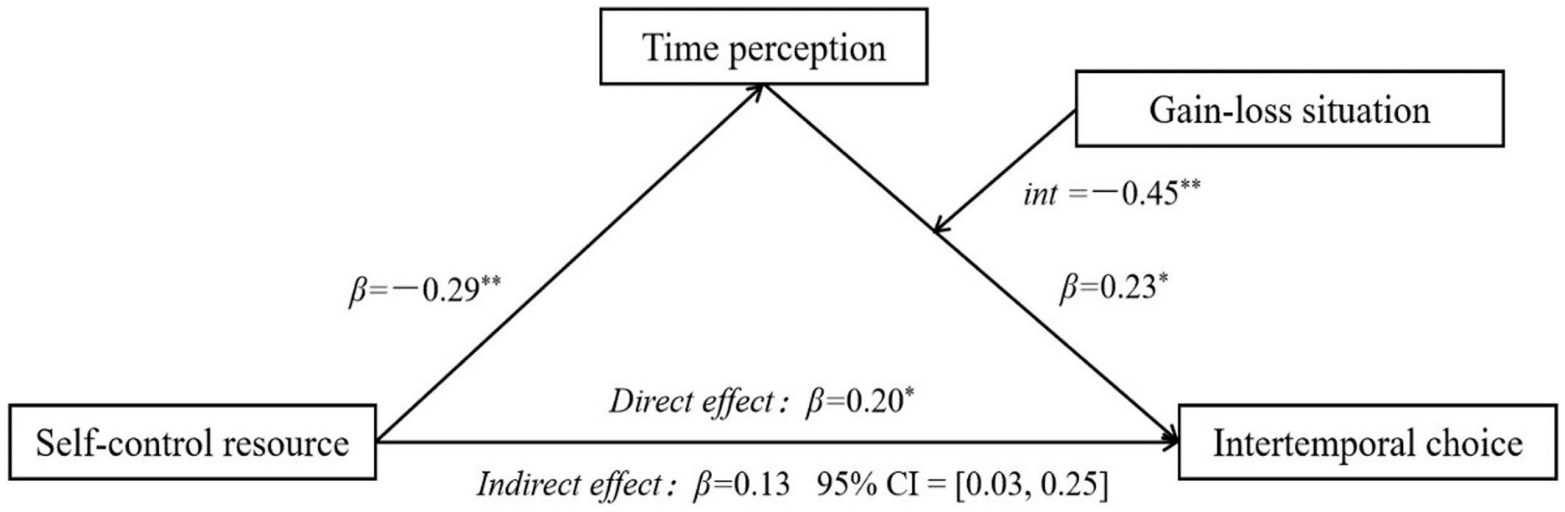
Mediation moderation model. ^*^p < 0.05, ^**^p < 0.01.

The results revealed that in the gain situation, time perception significantly and positively predicted intertemporal choices [*β =* 0.45, *t =* 3.72, *p* < 0.001, 95% CI *=* (0.21, 0.69)]. By contrast, in the loss situation, the predictive effect of time perception on intertemporal choices was nonsignificant [*β =* −0.001, *t =* −0.007, *p* = 0.994, 95% CI *=* (−0.24, 0.24)]. We further investigated the conditional indirect effect of time perception under different contexts. The results revealed that the mediating effect of time perception varied with whether a loss or gain situation was presented. In the gain situation, the mediating effect of time perception was −0.13 [95% CI *=* (−0.25, −0.04); not including 0]. However, in a loss situation, the mediating effect of time perception was 0.001 [95% CI *=* (−0.07, 0.06); including 0]. These results indicate that the indirect effect of state self-control on intertemporal choices through time perception gradually weakened in the loss situation, indicating the situation had a mediating moderation effect. In summary, the moderated mediation model holds, with a moderated mediation index of 0.13 [95% CI *=* (−0.25, −0.04); not including 0; Figure 7].

### 3.3 Discussion of experiment 2

The results of Experiment 2 reveal that the depletion group had a higher rate of choosing immediate options in intertemporal choices than did the nondepletion group. This finding is consistent with that of a previous study (Suo et al., 2018). According to self-regulation theory (Verplanken and Sato, 2011), self-regulatory mechanisms can inhibit impulsive processes, and as individuals use more of their self-regulation resources, the inhibitory effect on impulsive processes weakens. Initially, individuals make a concerted effort to resist impulsive behaviors. However, in the present study, because the participants’ self-control resources were depleted due to their participation in the e-crossing task, they were more attracted to the idea of gaining immediate rewards in the subsequent intertemporal decision tasks and less willing to endure lengthy waiting times, leading them to make more impulsive choices. This finding is consistent with Hypothesis 1. Furthermore, the time overestimation group exhibited a higher rate of choosing immediate options in intertemporal choices than that of the underestimation group. The participants in both groups exhibited a lower preference for immediate options in intertemporal choices in gain situations than in loss situations. This finding is consistent with the results of Experiment 1.

Our results reveal that self-control resources not only exerted a direct effect on intertemporal decision-making but also exerted an indirect effect by negatively predicting time perception. A previous study reported that individuals tend to overestimate time under conditions of ego depletion (Zhang et al., 2019). In contrast to these finding, those of the current study reveal that the degree of self-control depletion negatively predicted time perception. Because of fatigue, the participants in the self-control depletion group anticipated that they would complete subsequent tasks sooner, underestimating the required time. A previous study indicated that participants in its self-control depletion group tended to actually be willing to wait for a shorter period of time than that they estimated a task would take (Vohs and Schmeichel, 2003). The study revealed that time perception positively predicted the rate of choosing immediate options in intertemporal choices. Subjective time perception affects individuals’ estimation of objective time. Individuals with longer time perception are inclined to opt for immediate options because they overvalue the time cost of waiting (Huang et al., 2005; Droit-Volet and Dambrun, 2019). By contrast, individuals with shorter time perception tend to underestimate the time they must wait to receive delayed rewards.

Our results reveal that the gain–loss situation moderated the impact of time perception on intertemporal choices, providing support for Hypothesis 4. In a gain situation, individuals who overestimate time are more likely to overestimate the time cost of future gains and are inclined to choose immediate options. However, in a loss situation, their focus is narrowed because of negative emotions associated with losing money, which leads them to experience an absorption in feelings of sadness and makes it difficult for them to notice other factors. Individuals in a loss situation are influenced by the idea of “seizing the moment” and tend to choose immediate options.

## 4 Discussion

### 4.1 Effect of self-control on intertemporal choices

This study investigated the effect of self-control and time perception on intertemporal decision-making preferences in gain–loss situations through two experiments. The results of both experiments of this study indicated that the participants with higher levels of self-control, regardless of whether it was trait or state self-control, were more likely to opt for delayed options in intertemporal choices, whereas the participants with lower levels of self-control were more likely to opt for immediate options. This finding aligns with those of previous studies (He and Yan, 2015; Suo et al., 2018; Malkoc and Zauberman, 2019). Trait self-control is a stable personality trait that, in impulsive situations, can reduce impulsivity and enable people to thoughtfully consider long-term outcomes (Tangney et al., 2004; He et al., 2020). A study on the impulsivity of addiction determined that individuals with low levels of self-control in addiction cannot resist temptations and are unable to think with a long-term perspective, focusing only on immediate outcomes (Wilhelm et al., 2006). In real life, low levels of self-control are commonly observed in Internet addicts and drug users. In Experiment 2 of this study, we manipulated the participants’ self-control resources through an e-crossing task, and the results are consistent with those of Experiment 1. A previous study revealed that ego depletion causes individuals to engage in less complex thinking and to rely instead on intuitive processing of information. However, when self-control resources are abundant, individuals tend to consider multiple viable approaches to problem-solving and employ rational analysis for information processing (Pocheptsova et al., 2009). Therefore, individuals with abundant self-control resources tend to make decisions with a long-term perspective, favoring delayed options in intertemporal choices. By contrast, individuals with limited self-control resources tend to adopt a more impulsive approach, favoring immediate options in intertemporal choices. The theory of limited self-control resources posits that self-control resources are finite and gradually deplete when an individual employs cognitive, behavioral, or emotional self-control, and this ultimately leads to ego depletion and subsequent failure in self-control tasks (Baumeister, 2002).

### 4.2 Mediating role of time perception

In this study, the time overestimation group exhibited a greater tendency to choose immediate options than did the time underestimation group. This finding is consistent with those of previous studies (Wittmann and Paulus, 2008; Suo et al., 2018; Laube and van den Bos, 2020; Zhou et al., 2022). The results of Experiment 2 revealed that self-control resources negatively predicted time perception, indicating that the greater the ego-depletion level is, the more prone individuals are to underestimate time. The results of this study are inconsistent with those of previous studies. Studies have often reported that individuals with higher levels of self-control resource depletion tend to have fewer available self-control resources, and this prevents them from allocating sufficient resources to time perception, resulting in longer time estimates (Suo et al., 2014; Takahashi, 2016; Kim et al., 2017). However, another study determined that the processing of time information involves attention and memory (Ogden et al., 2011). Individuals must employ executive control when performing experimental tasks. Ego depletion impairs executive control, subsequently affecting the processing of time information and time perception and thus affecting intertemporal decision-making (Zhang et al., 2019). The research results found that in time estimation tasks, an increase in the depletion of self-control resources led to an underestimation of time. Individuals who exhibited self-depletion tendencies were more inclined to complete experimental tasks rapidly.

Experiment 1 did not indicate that time perception plays a mediating role in the effect of trait self-control on intertemporal choices. This finding may be attributed to both trait self-control and time perception being stable characteristics. After these two traits develop, they cannot be easily influenced or changed. According to the time perception model, the perception and assessment of time play a crucial role in intertemporal decision-making. From the perspective of time personality tendencies, subjective estimation of objective time varies among individuals. Individuals with longer time perception, causing them to overestimate the time required to obtain delayed rewards, perceive the risk associated with delaying a reward to be greater, leading them to be more inclined to choose immediate options. By contrast, individuals with shorter time perception tend to choose delayed rewards.

### 4.3 Moderating effect of gain and loss situation

The findings of this study revealed that the participants were more likely to choose immediate options in the loss situation than in the gain situation. This finding is consistent with those of previous studies. Previous research has indicated that the underlying mechanisms of intertemporal choices involving gains and losses are not symmetrical. For example, a sign effect exists, where the attractiveness of gains decreases more rapidly than the aversion to losses does, resulting in a phenomenon in which the discount rate for losses is smaller than that for gains (Tanaka et al., 2014; Jiang and Liu, 2021). Furthermore, the current study determined that in the context of self-control resources affecting intertemporal choices, time perception and gain–loss situations have moderating mediating effects. In particular, time perception positively predicts the immediate choice rate in gain situations, whereas the predictive effect of time perception on the immediate choice rate is nonsignificant in loss situations. This finding can be explained from two perspectives. On the one hand, the decision-makers not only compare the outcomes of different options but also assess associated risks when making intertemporal decisions (Kahneman and Lovallo, 1993; Weber et al., 2002). When people are presented with gain–loss situations involving the same amount of money, the pain of loss is greater than the pleasure of gain. Thus, individuals perceive higher levels of influence and risk in loss situations, leading them to make more cautious choices (Tong et al., 2012). On the other hand, Pei et al. (2022) suggested that this could be attributed to different emotions induced by various situations. In gain situations, individuals are presented with positive information, whereas in loss situations, individuals anticipate having negative and painful emotional experiences. Because people self-protect, they instinctively tend to reject situations that result in painful experiences. Individuals who consistently chose immediate options in loss situations revealed that this phenomenon was often related to individuals’ economic circumstances and the psychological burden associated with disliking owing money to others. This may explain why the majority of the participants in our study chose to repay the loss as soon as possible.

### 4.4 Limitations and directions for future research

This study provides novel findings on the interplay of trait and state self-control and time perception and how it influences the rates of selecting immediate options in both gain and loss situations by conducting an intertemporal choice task. This study has several limitations. Firstly, there are limitations in the version of the intertemporal choice task used in this study. Secondly, the primary focus of our investigation was on the rate of choosing immediate options, and participants were informed at the beginning of the experiment to base their decisions on their genuine feelings, with no time constraints, there exists considerable individual variability in reaction times. Consequently, the current study did not extensively explore the impact of reaction times in data analysis. In future research, we plan to address this limitation by refining the experimental design. We will pay more careful attention to the consideration of reaction times in intertemporal decision-making under ego depletion conditions. Thirdly, the study lacked a coefficient of stability for the time duration replication task. Subsequent research placed increased emphasis on the test–retest reliability of the time duration replication task to ensure the rigor and reliability of study results. Finally, considering the ongoing debate regarding the existence of the ego depletion effect (Hagger et al., 2016), in future studies, we plan to conduct more rigorous manipulation checks, such as assessing ego depletion levels after participants undergo different numbers of trials in intertemporal decision-making tasks.

## 5 Conclusion

Intertemporal choices and the ability to make objective and rational judgments regarding these choices are ubiquitous in daily decision-making. However, factors such as time, emotions, and energy may introduce cognitive biases that influence intertemporal decision-making behaviors. The current study revealed that individuals with higher levels of self-control, regardless of whether it was state or trait self-control, tended to prefer larger, delayed rewards, whereas those with lower levels of trait self-control were more inclined to choose smaller, immediate rewards. In addition, this study revealed that state self-control resources not only directly predict intertemporal choices but also indirectly predict such choices through the mediating effect of time perception. Finally, the study indicated that the gain–loss situation moderates the effect of time perception on intertemporal choices. In the gain situation, individuals in the time overestimation group were more likely to favor immediate options than were those in the time underestimation group. However, in the loss situation, no significant difference in intertemporal decision outcomes were noted between the two groups.

## Data availability statement

The original contributions presented in the study are included in the article/supplementary material, further inquiries can be directed to the corresponding author.

## Ethics statement

The studies involving humans were approved by the local Ethics Committee of Hunan Normal University. The studies were conducted in accordance with the local legislation and institutional requirements. The participants provided their written informed consent to participate in this study.

## Author contributions

WQ: Writing – review & editing, Funding acquisition, Project administration, Supervision. YY: Writing – original draft, Writing – review & editing, Conceptualization, Data curation, Investigation, Methodology. MZ: Writing – original draft, Conceptualization, Data curation, Software. WF: Writing – review & editing, Formal analysis, Validation, Visualization.
